# MRI volumetric changes in hippocampal subfields in psychosis: a protocol for a systematic review and meta-analysis

**DOI:** 10.1186/s13643-022-01916-5

**Published:** 2022-03-15

**Authors:** Anurag Nasa, Olivia Mosley, Elena Roman, Allison Kelliher, Caoimhe Gaughan, Kirk J. Levins, David Coppinger, Erik O’Hanlon, Mary Cannon, Darren William Roddy

**Affiliations:** 1 Department of Psychiatry, Trinity College Institute of Neuroscience, Lloyd Building, Trinity College Dublin, Dublin 2, Ireland; 2 Department of Anaesthesiology, Intensive Care and Pain Medicine, St. Vincent’s University Hospital, Dublin 4, Ireland; 3 Department of Psychiatry, Royal College of Surgeons in Ireland, Dublin 2, Ireland; 4 Department of Physiology, School of Medicine, University College Dublin, Dublin 4, Ireland

**Keywords:** Magnetic resonance imaging, Hippocampus, Psychosis, Cornu ammonis, Subiculum, Systematic review, Meta-analysis

## Abstract

**Background:**

The hippocampus has for long been known for its ability to form new, declarative memory. However, emerging findings across conditions in the psychosis spectrum also implicate its role in emotional regulation. Systematic reviews have demonstrated consistent volume atrophic changes in the hippocampus. The aim of the systematic review and metanalysis which will follow from this protocol will be to investigate the volume-based neuroimaging findings across each of the subfields of the hippocampus in psychosis independent of diagnosis.

**Methods:**

Volume changes across subfields of the hippocampus in psychotic illnesses will be assessed by systematic review following the Preferred Reporting Items for Systematic Reviews and Meta-Analyses (PRISMA). MRI neuroimaging studies of patients with a definitive diagnosis of psychosis (including brief pre-diagnostic states) will be included. Studies lacking adequate controls, illicit drug use, medical psychosis, history of other significant psychiatric comorbidities, or emphasis on age groups above 65 or below 16 will be excluded. Subfields investigated will include the CA1, CA2/3, CA4, subiculum, presubiculum, parasubiculum, dentate gyrus, stratum, molecular layer, granular cell layer, entorhinal cortex, and fimbria. Two people will independently screen abstracts from the output of the search to select suitable studies. This will be followed by the two reviewers performing a full-text review of the studies which were selected based on suitable abstracts. One reviewer will independently perform all the data extraction, and another reviewer will then systemically check all the extracted information using the original articles to ensure accuracy. Statistical analysis will be performed using the metafor and meta-packages in R Studio with the application of the random-effects model.

**Discussion:**

This study will provide insight into the volumetric changes in psychosis of the subfields of the hippocampus, independent of diagnosis. This may shed light on the intricate neural pathology which encompasses psychosis and will open avenues for further exploration of the structures identified as potential drivers of volume change.

**Systematic review registration:**

PROSPERO CRD42020199558

## Background

### Psychosis

Psychosis is a broad term that encompasses symptoms related to a change in perception of reality. It is found in many psychiatric, neurologic, neurodevelopmental, and medical conditions [50]. The diseases in which psychosis is considered a core symptom are outlined in the Schizophrenia Spectrum and Other Psychotic Disorders section of the Diagnostic and Statistical Manual of Mental Disorders: Fifth Edition (DSM-5) [7]. Such disorders include schizotypal (personality) disorder, delusional disorder, brief psychotic disorder, schizophreniform disorder, schizophrenia, schizoaffective disorder, substance/medication-induced psychotic disorder, and psychotic disorder due to another medical condition and various catatonias [7]. DSM-5 states that these disorders “are defined by abnormalities in one or more of the following five domains: delusions, hallucinations, disorganized thinking (speech), grossly disorganized or abnormal motor behaviour (including catatonia), and negative symptoms” [50]. Furthermore, psychosis as defined by these abnormalities is also found as a qualifier in other non-primarily psychotic conditions, including major depressive disorder, bipolar affective disorder, and post-traumatic stress disorder. Schizophrenia is considered the archetypal psychotic disorder with an incidence of 15.2 per 100,000 (McGrath). However, psychotic symptoms are not confined to schizophrenia and 1.5 to 3.5% of people will meet diagnostic criteria for a psychotic disorder at some point in their lifetime (Van os). Many brain areas have been implicated in psychosis with the hippocampus being one of the regions most consistently identified in studies.

### The hippocampus and its substructures

The hippocampus, located deep within the medial temporal lobe, is one of the most investigated structures in the brain [16]. Initial research concentrated on its role in forming new, declarative memories [19, 41], and spatial organization [40]; however, as findings emerged of hippocampal involvement across neuropsychiatric disorders, the hippocampal role in emotional regulation became a focus for research [11, 12, 15, 33, 39]. The hippocampus is structurally plastic throughout life and is one of the few areas that can undergo adult neurogenesis in the brain [32]. As an extension underneath the cerebral cortex, it lies along the floor of the lateral ventricle and forms an integral hub of the limbic system [5]. The hippocampus has an S-shaped structure that consists of two histologically distinct parts separated by the hippocampal fissure: the hippocampus proper or cornu ammonis (CA) region and the dentate gyrus [5]. The CA regions lie on the subiculum, which extends to the entorhinal cortex. The CA, dentate, and subiculum together form a unit called the hippocampal formation or “hippocampus” [45]. A unique feature of the structure is the largely (but not exclusively) unidirectional information flow through the hippocampal system [4]. External sensory and internal cortical/subcortical information funnels through the entorhinal cortex to enter the hippocampus via the dentate granule cells. The dentate can be considered the pre-processor of inputs into the hippocampus [29], playing a critical role in mediating some of the higher brain functions of the hippocampus, namely memory, learning, and spatial coding [2]. It is also the only area within the hippocampus where neurogenesis occurs [1], allowing for new neurons to be generated throughout life. Dentate neurogenesis dysfunction has been implicated in some psychiatric disorders [10, 48, 49]. From the dentate, information flows to the CA regions. The CA region is divided into four regions (CA1-3), with CA4 lying within the hilum of the dentate and considered functionally part of this structure [3]. The dentate to the CA3 pathway is known as the mossy fiber pathway. The CA3 region has been implicated in spatial awareness [40] and as a hippocampal pacemaker coordinating alertness and encoding [59]. Aberrant CA3 neuronal activity has been shown in schizophrenia [8]. CA2 is a relatively small and indistinct area interposed between the larger CA3 and CA1 and appears to have unique connectivity with the amygdala and HPA axis [42] and has suggested a role in social cognition [27]. Smaller CA2 regions have been found in the post-mortem brains of schizophrenia patients [38]. CA1 is by far the largest hippocampal subfield [58] and has a role in autobiographical memory [31] with pathology being shown in dementia [37]. Finally, information flows from the CA regions to the subiculum. The human subicular region is divided into the subiculum, presubiculum, and parasubiculum [60]. The pre- and parasubiculum are more parahippocampal in origin and function and may be considered input hubs of the entorhinal cortex [61]. With the CA1 region, the subiculum proper may be considered the output region of the hippocampus, with both structures returning information to the deep entorhinal cortex and directly out from the hippocampus.

### The hippocampus and psychosis

Psychosis is associated with early life trauma. Studies have found that early life stress may result in microstructural hippocampal changes such as reduced neurogenesis and dendritogenesis [48], leading to reduced hippocampal volumes [20]. Early life adversity is thought to interact with inherent genetic vulnerabilities resulting in hippocampal morphological changes and dysfunctional information processing [14]. The hippocampus is the major hub integrating memory and emotion and dispersing information throughout the limbic system to and from the amygdala, thalamic, cingulate, and frontal regions [44, 47, 57]. Hippocampal dysfunction has been suggested as a potential aetiological factor for psychosis, which features disordered information processing [14].

Hippocampal involvement across the psychosis spectrum has been demonstrated with smaller hippocampal volumes consistently shown in psychotic disorders such as schizophrenia [62] and schizoaffective disorder [6]. Patients with bipolar affective disorder with psychosis appear to show smaller hippocampi [52] but not bipolar patients without psychosis [36]. Conversely, although depression is consistently associated with smaller hippocampal volumes [46], depression with psychosis appears to show little association with hippocampal volume [30]. “Premorbid” and “at-risk” psychotic states where individuals experience brief or limited psychotic symptoms (and often go on to develop true psychotic conditions) are also associated with smaller hippocampal volumes [11].

### Magnetic resonance imaging of hippocampal subfields and psychosis

Recent hardware advances in MRI such as increased fields strengths (3 T, 4 T, 7 T, and higher), improved acquisition protocols, and the development of sophisticated pre-processing techniques combined with improved computational power have allowed greater accuracy and speed in quantifying hippocampal volumes. Specifically, the increased resolution achieved through these advances has allowed researchers to consistently quantify hippocampal volumes at the substructure level (e.g., CA1-4, subiculum, dentate). The advent of automated segmentation techniques based on detailed high-resolution atlases has facilitated the measurement of hippocampal subfields in larger datasets. All hippocampal regions have been shown by various studies to be smaller in psychotic disorders. Although there have been disorder-specific reviews of hippocampal subfields in schizophrenia [28] and bipolar disorder [24], a comprehensive review and meta-analysis of hippocampal subfields focusing on the presence of psychosis rather than specific diagnoses has yet to be published. A common hippocampal subfield signature across the entire psychotic spectrum may provide deeper insights into the aetiology of the symptoms of psychosis and potentially reveal new common therapeutic targets for psychosis.

## Methods

### Search strategy

Online databases will be searched for relevant articles. The databases examined will include PubMed, Google Scholar, MEDLINE, and EMBASE, from where articles will be systematically assessed to identify those relevant to our hypothesis. The search performed was as follows: [(MRI OR Magnetic Resonance Imaging) AND (Psychosis OR Brief Limited Intermittent Psychotic Symptoms) AND (Schizophrenia OR Bipolar Disorder OR Schizoaffective OR Depression OR delusional disorder OR Brief psychotic disorder OR Schizophreniform disorder OR Medication-induced psychotic disorder OR hallucinations OR delusions OR thought disorder OR catatonia OR personality disorder)].

References from the output articles will also be checked, and articles that are pertinent to our study will also be incorporated into it. The search items will be rerun before publication to include newer studies that got added to the databases.

### Eligibility criterion

#### Inclusion criteria

The studies to be included in this review will encompass MRI neuroimaging studies of patients with a definitive diagnosis of psychosis and comparisons with healthy control participants. The studies that segmented the hippocampus’s subfields using either automatic or manual means will be included.

All the studies included will have been peer-reviewed. Although the search items were in English, we will include non-English studies, which we will get translated professionally, and contact the corresponding author with any confusions which may arise.

Studies with an emphasis on ages above 65 or below 16 will also be excluded. Incomplete or ambiguous information will be clarified by contacting the corresponding authors of the respective studies. In instances where the results from a study have been reported in more than one article, the results from the article with a greater sample size will be extracted.

#### Exclusion criteria

Studies lacking a control group will be excluded. Studies where illicit drug use is documented or those with a history of other significant psychiatric comorbidities will be excluded. We acknowledge that some studies may not document drug use. Studies with participants that had medical psychosis will also be excluded since psychiatric psychosis is the focus of this review.

Abstracts from the studies that are output from the search strategy will be screened by two researchers. The full text will then be reviewed by the two researchers separately. We will include case-control, cohort, cross-sectional studies, randomized control trials, and longitudinal studies. Inconsistencies or conflicts regarding the studies to be selected will be discussed and resolved with guidance from Dr. Roddy.

#### Data collection

One reviewer will independently perform all the data extraction, and another reviewer will then systemically check all the extracted information using the original articles to ensure accuracy. The information to be extracted includes the following:

1. Author and publication year
2. Cohort information (the specific psychotic illness(es) the study discusses)
3. Sample and descriptive demographic information (age, gender)
4. Age of onset, duration of illness, and duration of untreated psychosis
5. Diagnostic method and quantification criteria for psychosis with results, e.g., PANSS, BPRS, and SAPS+SANS
6. Whether the participants were medicated or not. If so, which medication class was used?
7. Software used for hippocampal subfield volume determination and tracing type, i.e., automatic or manual
8. Type of MRI scanner used and magnetic field strength
9. Volumetric information for each of the subfields in both hemispheres
10. Illicit drug use

### Hippocampal subfield volumes

Quite often in neuroimaging studies, the definition and segmentation of subfields are conducted using different methodologies and software: manual or automatic. Though the output from these should be very similar, we will be cautious of possible heterogeneity which may arise due to this by recording the software used in each instance, allowing for a possible explanation of heterogeneity if it arises during our analysis.

The subfield volume information we will extract will be that of the patients and controls within each of the studies. We will be extract volumes of the CA1, CA2, CA3, DG, CA4, Dentate Gyrus (DG), Subiculum, Presubiculum, and Parasubiculum. In instances where composite volumes are presented in the paper, those will be recorded. CA4-DG and CA2-3 are pairs that can be difficult to tell differentiate, particularly using automated software. Hence, we expect that we will frequently be recording composite volumes for those and will be running analysis on them as a composite.

### Meta-analysis

Statistical analysis will be conducted using the metaphor and meta-packages in R Studio 2020 (RStudio, PBC, Boston, MA; URL http://www.rstudio.com), which is an integrated development environment for R [54, 56]. Given the assumption of exchangeability in a random-effects model, it will be applied throughout our review to weight each study and control for potential heterogeneity [13, 43]. The potential heterogeneity we may encounter will be explored and include the software used for subfield segmentation, study design, duration of illness, type of psychotic disorder, age of onset, medication use, measurement on scales for psychosis, and magnetic field strength of the MRI scanner used. Cohen’s *d* statistic or Hedges’ unbiased *g* will be computed, as appropriate, for an effect size of the difference between means of the patient and control groups [22]. Potential type 1 inflation errors will be addressed using conservative correction measures such as false discovery rate [9] or Bonferroni [51].

### Meta-regression

Meta-regression will be employed for the assessment of our secondary and tertiary hypotheses that subfield volumes change with the severity of psychosis and with the duration of illness. The first regression analysis performed will examine the relationship between standardized mean differences (SMD) of the measurements on psychosis scales and Cohen’s *d* effect sizes for volume change effects in each of the subfields. Another regression analysis will then examine the relationship between duration of illness and volume change effects in each of the subfields. These analyses will be performed using SPSS-26 (IBM SPSS Statistics 26 for Windows 10).

### Between-study heterogeneity

Assessment of between-study heterogeneity will be conducted using Cochran’s *Q*, and the degree of heterogeneity will be quantified using the *I*^2^ statistic [26]. This will give us the percentage of variability that is due to differences between studies compared to sampling variance. The interpretation of these *I*^2^ values will be 0.25 = low, 0.5 moderate, and 0.75 = high. The significance threshold for establishing the studies are heterogenous will be 0.1, as a higher alpha level is recommended using Cochran’s *Q* test to determine statistical heterogeneity when few studies are included or event rates are low [53]. In instances where the *Q*-statistic is significant, Galbraith plots will be produced to supplement forest plots in determining the studies which have the largest influence on increasing the heterogeneity [21].

### Bias

Quality of evidence will be evaluated using the Grading of Recommendations, Assessment, Development and Evaluations (GRADE) criteria with five domains of evidence being assessed (risk of bias, imprecision, inconsistency, indirectness, and publication bias) each according to four levels of quality (very low, low, moderate, and high) [23]. Publication bias and small-study effects will be important features addressed as part of this review. These occur when mostly the significant findings get published [17]. Small study effect refers to phenomena where studies with smaller samples, and less power, tend to report larger effect sizes [55]. These will be verified by visual inspection of funnel plots and assessed using the Eggers test [18]. Studies that do not appear adequately robust will be eliminated from the quantitative meta-analysis but may be documented narratively throughout the paper. The risk of bias in observational studies (i.e., flaws in the study design, conduct, or analysis) will be assessed using Newcastle-Ottawa Scale, where studies will be graded according to three quality outcomes: group selection, group comparability, and outcome [35]. The Cochrane Risk of Bias tool will be used to assess bias in randomized control trials [25].

### Data synthesis

This study will obtain clinical, demographic, and methodological variants. A forest plot will be used to synthesize the total number of participants, studies, subfield volumes with mean differences, 95% confidence intervals, *p* values, and *I*^2^ statistics in graphical form [34]. A sub-type analysis will be performed for the different study designs, tracking means (automatic vs manual), software used, and for the different subfields of the hippocampus. If a meta-analytical approach is not feasible based on heterogeneity and sample sizes, we plan to summarize the findings as a narrative systematic review.

### Strengths and limitations

Important strengths of this study will include the first detailed characterization of the hippocampal subfields in psychotic disorders and an evidence base that encompasses varying study designs. Methodological strengths include the systematic nature of the data acquisition and collection, according to PRISMA guidelines and robust statistical analysis. Limitations include the potential heterogeneity in methodologies for participant recruitment, subfield segmentation, and software packages used.

## Conclusion

This is a protocol for a systematic review and meta-analysis to summarize findings from MRI neuroimaging studies of the hippocampal subfields in psychosis. While hippocampal atrophy has been well documented in prior studies, this study will provide another layer of specificity in outlining the subfields which drive that effect. Adjacent to this, it will seek whether the duration of illness and degree of psychosis impact these effects.

## Data Availability

Data sharing does not apply to this article as no datasets were generated or analyzed during the current study.
